# Predictors of Neurological Worsening after Resection of Spinal Meningiomas

**DOI:** 10.3390/cancers15225408

**Published:** 2023-11-14

**Authors:** Dragan Jankovic, Darius Kalasauskas, Ahmed Othman, Marc A. Brockmann, Clemens J. Sommer, Florian Ringel, Naureen Keric

**Affiliations:** 1 Department of Neurosurgery, University Medical Center, Johannes Gutenberg University Mainz, 55131 Mainz, Germany; dragan.medicine@gmail.com (D.J.); darius.kalasauskas@unimedizin-mainz.de (D.K.); naureen.keric@unimedizin-mainz.de (N.K.); 2 Department of Neuroradiology, University Medical Center, Johannes Gutenberg University Mainz, 55131 Mainz, Germany; ahmed.othman@unimedizin-mainz.de (A.O.); marc.brockmann@uni-mainz.de (M.A.B.); 3 Institute of Neuropathology, University Medical Center, Johannes Gutenberg University Mainz, 55131 Mainz, Germany; clemens.sommer@unimedizin-mainz.de

**Keywords:** meningioma, outcome, spine, surgery

## Abstract

**Simple Summary:**

Despite the benign nature of these tumors, spinal meningiomas can cause significant neurological damage via compression of the spinal cord. In this study, we found that neurological function improves in a significant proportion of patients after surgery. Preoperative Frankel grade was a significant predictor of postoperative neurological worsening. Cross-section area measurements on MRI scans are not associated with early postoperative outcomes.

**Abstract:**

Background: Due to the slow-growing nature of spinal meningiomas, they are mostly asymptomatic for a long time, and become symptomatic after the compression of the spinal cord or nerve roots. The aim of this study was to identify predictors for a poor clinical outcome after the surgical resection of spinal meningiomas and thereby to allow a preoperative identification of high-risk spinal meningiomas. Methods: Data acquisition was conducted as a single-center retrospective analysis. From 1 January 2004 to 31 December 2019, 121 patients who underwent surgical resection of a spinal meningioma were reviewed. Clinical and radiological data (such as tumor size, location, occupation ratio of the spinal canal, and the degree of spinal cord compression) were assessed. The functional clinical findings of the patients were recorded using the Karnofsky Performance Score, modified McCormick scale, and Frankel scale preoperatively, at discharge, and 3–6 months after surgery. Results: The mean patient age was 66 ± 13 years. A total of 104 (86%) patients were female and 17 (14%) were male. The thoracic spine (68%) was the most common location, followed by the cervical (29%) and lumbar (3%) spine. Preoperatively, 11.7% of patients were categorized as McCormick 1, 35.8% as 2, 39.2% as 3, 11.7% as 4, and 1.7% as 5. The neurological function of the patients with a functional deficit prior to surgery improved in 46% of the patients, remained unchanged in 52%, and worsened in 2% at discharge. At early follow-up, the proportions were 54%, 28%, and 5%, respectively. Preoperative Frankel scale was a significant predictor of a postoperative deterioration. Patients with Frankel score A to C preoperatively had a 9.2 times higher chance of clinical deterioration postoperatively (OR = 9.16). We found that the Frankel scale weakly correlated with the degree of spinal cord compression. In this study, other radiological parameters, such as the degree of cord compression and spinal canal occupation ratio, did not show a significant effect on the outcome. Conclusions: Surgery of intraspinal meningiomas can be considered safe. Neurological function improves in a large proportion of patients after surgery. However, a relevant preoperative deficit according to the Frankel scale (grade A–C) was a significant predictor of a postoperative neurological deterioration.

## 1. Introduction

Spinal meningiomas (SMs) are the most common intradural extramedullary tumors, accounting for 25–45% of all primary intradural spinal tumors and 7.9–12% of all meningiomas [1,2,3]. They occur with an age-adjusted incidence of 0.33 per 100,000 inhabitants [4]. The incidence increases with age, and most meningiomas occur from the fifth decade of life [5]. In SMs, the female-to-male ratio is approximately 5:1, compared to a 2:1 ratio in intracranial meningiomas [6,7,8].

They are typically solitary, well-circumscribed neoplasms not invading other adjacent tissues [9]. Histologically, the majority of SMs are CNS grade 1, according to the classification of the World Health Organization [10]. However, they can also appear as CNS grade 2 and grade 3, which are associated with a more aggressive growth and a high risk of recurrence after resection [10].

Surgical resection is the first choice of treatment and is predominantly performed via a posterior spinal approach and hemilaminectomies. Although the postoperative course may be heterogeneous, most patients show satisfactory results with neurological improvement after surgery. Their mortality is low, and it is mostly associated with older age and severe comorbidities [11].

However, despite the benign nature of these tumors, they can cause significant neurological damage via compression of the spinal cord. Furthermore, for a small subgroup of patients, the postoperative outcome is unfavorable and associated with new neurological deficits. Therefore, the present study aimed to identify preoperative risk factors for an unfavorable outcome and, thereby, to identify high-risk spinal meningiomas.

## 2. Patients and Methods

The medical records and MRIs of 135 consecutive patients undergoing surgical resection of SM in our department between January 2004 and December 2021 were retrospectively reviewed. The inclusion and exclusion criteria were defined prior to the start of the study.

The inclusion criteria were (1) age > 18 years, (2) surgical treatment of SM, (3) availability of preoperative MRI (T1-weighted with contrast agent and T2-weighted sequences), and (4) clinical outcome data until at least 3 months after surgery.

The exclusion criteria were (1) patients with only one postoperative follow-up after the initial surgery and (2) incomplete medical documentation.

A total of 14 patients were excluded according to the inclusion and exclusion criteria due to incomplete documentation of medical data. Thus, 121 patients were included in the complete data processing.

Baseline characteristics, including sex, age, patient history, and Charlson comorbidity index, were assessed [12].

Based on neuroradiological images, tumor location was divided by spinal level into four categories: cervical, thoracic, lumbar, and sacral. Further, we classified SM by attachment to the dura mater into ventral, dorsal, lateral, dorsolateral, and ventrolateral.

In addition, intraoperative data, such as the operative approach, duration of the operation, and intraoperative blood loss, were documented. Histopathological data, such as tumor grade according to the WHO, histological tumor subtype, and Ki-67 index, were taken from the neuropathological records.

The functional clinical findings of the patients were recorded using the Karnofsky Performance Score (KPS), modified McCormick scale (Table 1), and Frankel scale (Table 2) at admission to the hospital, discharge from the hospital, and the first postoperative and last visit to our clinic [13,14,15].

Early follow-up is defined as a follow-up between 3 and 6 months after surgery, whereas late follow-up represents the patient’s last visit to the outpatient clinic.

## 3. Radiologic Assessment

A quantitative assessment of the tumor was performed using MR images. Tumor volume was estimated using sagittal and axial T1-weighted MRI sequences with the following formula:

Volume (mm^3^) = (π × height × width × depth)/6

Height (H) was measured on sagittal images, whereas width (W) and depth (D) were measured on axial images. All measurements were recorded in millimeters.

**Figure 1:**
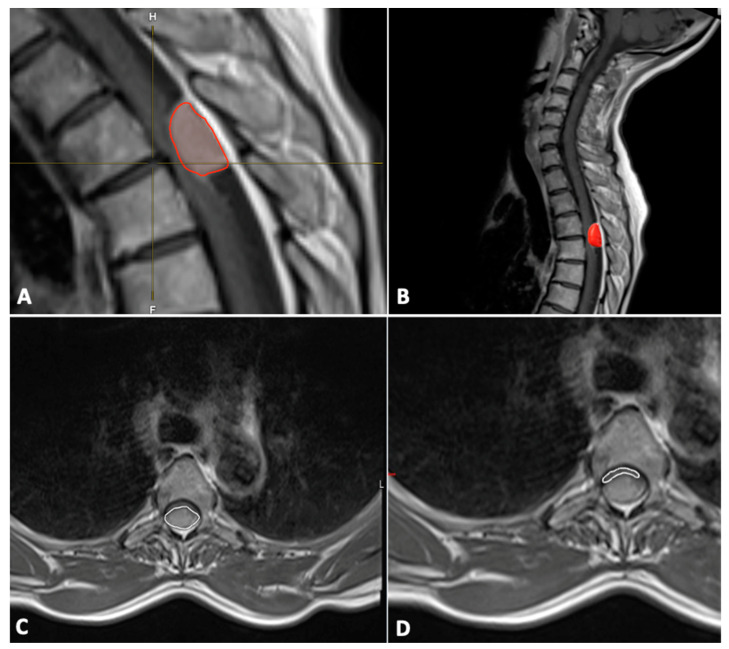
Tumor (red frame in the image) volume measurements with Brainlab Elements^®^ version 3.1.0 (**A**,**B**); cross-section of spinal meningioma (**C**) and spinal cord (**D**).

In addition, the tumor volume (mm^3^) was also segmented manually using Brainlab Elements^®^ version 3.1.0 (Brainlab AG, Munich, Germany) using axial and sagittal contrast-enhanced T1-weighted images (Figure 1).

The cross-sectional area (mm^2^) of the spinal canal, spinal cord, and tumor were measured on the axial T1-weighted contrast-enhanced MR image at the level of maximal tumor occupancy of the spinal canal (Figure 1).

The occupation ratio of the tumor in the spinal canal was calculated using the following formula:

Occupancy ratio (%) = tumor area (mm^2^)/spinal canal area (mm^2^) × 100

The degree of compression of the spinal cord by the tumor was calculated on the axial T2-weighted MR images using the following formula:

Spinal cord compression ratio (%) = 100 − (area of spinal cord at maximum compression/[spinal cord area above + spinal cord area below]/2) × 100

## 4. Statistical Analysis

Data analysis was performed using the statistics software SPSS (Version 16.0, SPSS Inc., Chicago, IL, USA). Categorical data are represented by absolute and relative counts.

Differences in normally distributed numerical variables between two independent groups were tested using Student’s *t*-test. The Mann–Whitney *U*-test was chosen to compare continuous variables as the data were mainly not normally distributed. 

Logistic regression was used to assess the influence of multiple factors on treatment outcome. Results with *p* < 0.05 were considered statistically significant.

## 5. Results

A total of 121 patients were included in this study. The mean age of all patients was 66 ± 13 years (range, 29–88 years), and 86% of 121 patients were females. The detailed patient characteristics are provided in Table 3.

### 5.1. Symptoms

The duration of the symptoms prior to the first presentation varied. Five patients (4.1%) showed acute symptoms within the 2 weeks prior to the diagnosis of SM. In total, 32 patients (26.4%) had medium-term symptoms ranging from 3 to 8 weeks, and 78 (64.5%) had longer-standing symptoms for more than 8 weeks.

The largest number of patients presented with sensory deficits as the leading symptom (present in 68.6% [*n* = 82] of the patients), followed by ataxia (in 55.4% [*n* = 65] of the patients) and pain (in 52.1% [*n* = 62] of the patients). Motor deficits were present in 47.9% (*n* = 56) of the patients, whereas hyperreflexia was present in 33.1% (*n* = 39). A positive Babinski reflex was documented in 16.5% (*n* = 20) of the patients, and 7.4% (*n* = 9) had bladder or rectum disorders. 

In total, 19 percent of the patients had a monosymptomatic initial disease manifestation, whereas 78.5% (*n* = 95) of the patients had two or more symptoms at initial presentation. Three patients had no symptoms, and all three had SM as an incidental finding. In these three patients, the decision on surgical treatment was made based on tumor growth during follow-up.

### 5.2. Location and Dural Attachment

Among the patients, about two-thirds of the SMs (67.2%, *n* = 82) were located in the thoracic spine, followed by the cervical spine (29.8%, *n* = 36) and lumbar spine (2.5%, *n* = 3).

A dorsal or posterior dural attachment was the most common in the examined group. It was present in 39.4% (*n* = 48) of the patients. The attachment was located anteriorly in 31.2% (*n* = 38) of the patients and located laterally in 19.6% (*n* = 24). In 5.7% (*n* = 7) of the patients, the dural attachment was anterolateral, and it was posterolateral in the remaining 4.1%.

Multiple meningiomas were present in 4.1% (*n* = 5) of the patients.

### 5.3. Surgical Treatment, Complications, and Recurrence

In 96% of the patients, the tumor was resected via a posterior approach, followed by a lateral approach in 2.4% and an anterior approach in 1.6%. The mean operation time was 183 ± 68 (range: 92–430 min) minutes. The mean intraoperative blood loss was 402.8 ± 205 mL.

Perioperative complications occurred in 5.6% of the patients. The most common postoperative complications were epidural hematoma (three patients), followed by CSF leak (in two patients). All five patients underwent a surgical revision, and no further complications occurred thereafter. The mortality rate was 0%. 

Four patients were operated on for a recurrent tumor; i.e., recurrence was present in 3.3% (*n* = 4) of the patients. The mean time until recurrence was 52 months.

Two patients with recurrence presented with a calcified tumor and one patient with SM recurrence presented with multiple spinal and intracranial meningiomas, whereas in the fourth recurrent case, a partial resection was initially performed, and the regrowth of the tumor was recorded at follow-up.

In 93.4% (*n* = 113) of the patients, the tumor tissue was pathohistologically classified as a CNS WHO Grade 1 meningioma, while 4.1% (*n* = 5) of the patients had a CNS WHO Grade 2. The most common histological subtype of CNS WHO Grade 1 meningiomas was psammomatous meningioma (52.1%), followed by transitional (20.7%) meningothelial (16.5%).

The average Ki-67 index in our cohort was 3.7 (range: 2–10%). Among all WHO CNS Grade 2 spinal meningiomas, the Ki-67 index was 10%.

The patient data and clinical parameters are summarized in Table 3.

### 5.4. Radiological Measurements

The mean tumor volume was 1032.1 mm^3^, with a minimum value of 78.4 mm^3^ and a maximum of 3126.5 mm^3^. Additional measurements are provided in Table 4. Myelopathy was present in 63.6% (*n* = 77) of the patients, whereas syringomyelia was visible on MRI scans in 4.1% (*n* = 5).

### 5.5. Analysis of Preoperative Functional Status and Postoperative Outcome

**Figure 2:**
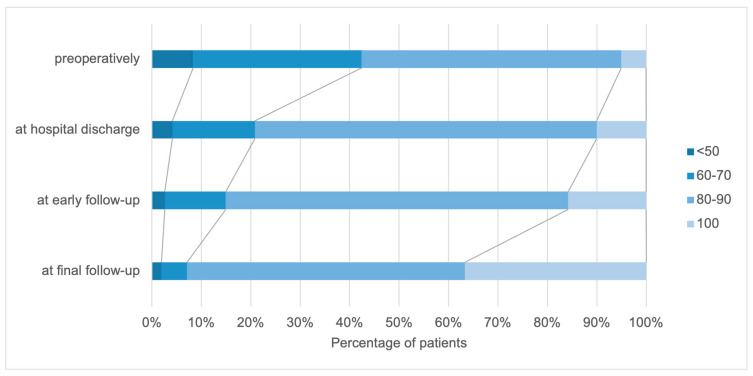
Distribution of patients according to the (KPS) preoperatively, at hospital discharge, and at early and final follow–up.

In the examined group, KPS values between 40% and 100% were found at the time of admission when collecting the KPS (Figure 2). The mean KPS was 76 ± 13. More than 50% of the patients had a KPS of 80 or higher upon admission to the hospital, meaning they were able to work and function normally.

Neurological improvement was recorded in a considerable proportion of patients directly after the operation. Comparing the preoperative KPS and the KPS at the time of hospital discharge, the number of patients without symptoms and with mild functional impairment increased: there was a 5% increase in the proportion in the KPS 100% group and a 16% increase in the KPS 80–90% group. The functional performance continued to improve in the short-term as well as the long-term follow-up. At the last visit, 92 patients had a satisfactory KPS score, with KPS scores of 100 in 36.2% of the patients compared to the preoperative scores. Deterioration in the KPS score of the patients’ functional status was noted in five patients. 

A similar trend was observed in the analysis of the McCormick and Frankel scales. The preoperative examinations resulted in an average McCormick scale value of 2.6. The mean McCormick scale value at hospital discharge was 2.09. Preoperatively, 11.7% of the patients were categorized as McCormick 1, 35.8% as McCormick 2, 39.2% as McCormick 3, 11.7% as McCormick 4, and 1.7% as McCormick 5. Postoperatively, 22.5% of the patients were categorized as McCormick 1, 52.5% as McCormick 2, 19.7% as McCormick 3, 5% as McCormick 4, and 0.8% as McCormick 5.

**Figure 3:**
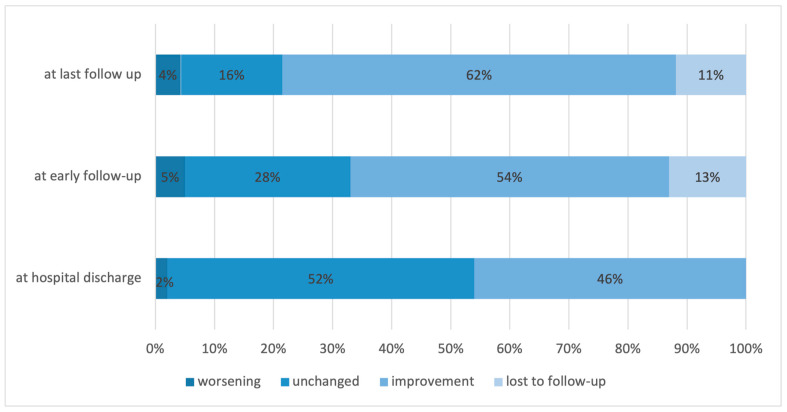
Distribution of patients based on the change in their neurological function at hospital discharge, at early and final follow-up.

Neurological function improved in 46.2%, remained unchanged in 52.4%, and worsened in 2.4%. At the early follow-up, the proportions were 54.3%, 28.3%, and 5.2%, respectively (Figure 3).

Preoperatively, 1.7% of the patients were categorized as Frankel grades A and B, whereas 34.2% of the patients were Frankel grade C. Frankel grade D was the most common preoperatively and 54.2% of the patients received this grade, whereas 8.3% of the patients were categorized as Frankel grade E. When comparing the different follow-up intervals, a continuous improvement was observed in all Frankel grades.

In the entire examination interval from before the operation to the last follow-up, an improvement in the patient’s functional status was determined in 75.5% of the patients. Comparing the Frankel scale values preoperatively and at the last visit, 59.4% of the patients improved by one grade, whereas 16.1% improved by two grades.

**Figure 4:**
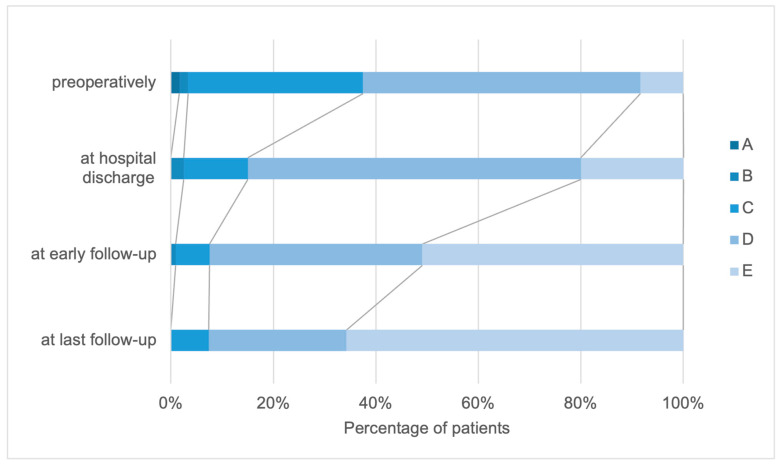
Distribution of patients according to the Frankel scale (A–E) preoperatively, at hospital discharge, and at early and final follow-up.

In 22.3% of the patients, the Frankel scale remained unchanged throughout the examination interval, whereas the functional status according to the Frankel scale deteriorated in 2.83% of the patients (Figure 4).

### 5.6. Correlation between Radiological Parameters and Functional Outcomes

We found significant differences in the tumor volume (*p* < 0.001), tumor cross-section (*p* < 0.001), and occupancy ratio (*p* = 0.03) according to the preoperative McCormick scale (Table 5). 

There were significant differences in the tumor volume (*p* = 0.007), tumor cross-section (*p* = 0.03), and occupancy ratio (*p* = 0.03) associated with different Frankel grades, but there were no significant differences in the spinal cord cross-section and the degree of cord compression (Table 6).

We used Spearman’s correlation coefficient to assess the association of the Frankel grade with the degree of cord compression, occupation ratio, surgical duration, and blood loss. We observed that there was a significant but weak negative relationship between the Frankel grade and the degree of cord compression (Rho = −0.228), operative time (Rho = −0.249), and blood loss (Rho = −0.222), and that there was no significant correlation with the occupation ratio (Table 7).

Multivariate logistic regression showed that a Frankel grade is a significant predictor for a worsening of neurological function. Patients with a worse Frankel scale (grade A–C) result when admitted to the hospital had a 9.16 times higher probability of a worsening of clinical symptoms (OR = 9.16), whereas tumor localization in the lower part of the thoracic spine was protective compared to the upper cervical part (OR = 0.28) (Table 8).

## 6. Discussion

The present study aimed to analyze the potential preoperative clinical and imaging factors associated with a postoperative neurological deterioration. Overall, our results show that the surgery of spinal meningiomas is safe, and that a significant proportion of patients experience an improvement in their functional performance before discharge. Further functional improvement was observed in the short-term and long-term follow-up. Only a small number of patients suffered from a worsening of functional performance after surgery. Our study shows that the Frankel scale may be a good tool for assessing the risk of a postoperative worsening in patients with spinal meningiomas. Furthermore, although we observed a correlation between the degree of spinal cord compression by the tumor and preoperative deficits, cross-sectional area measurements were not associated with the early postoperative outcome.

El-Hajj et al. reported in a systematic review of 49 studies that the adjusted complication rate for the surgical resection of spinal meningiomas is 7.4% [16]. This is slightly higher than our results of 5.6%. The most common complication in the published review was CSF leakage, while in our study, epidural hematomas were the most common complication, followed by CSF leaks.

In addition, the authors of the systematic review reported that the cumulative complication rate of patients older than 70 years is 16.8%, which exceeds the complication rate of the overall cohort of spinal meningioma patients [16]. In our study, we could not associate age with a higher complication rate, which is similar to previous studies [8,17]. In contrast, Schwanke et al. stated that the majority of complications in their cohort occurred in older patients [18].

Furthermore, some studies reported that an anterior dural attachment was associated with a complication rate of 26.3%, with cervical spine tumors being associated with the majority of the complications. We did not find an association between dural attachment location and complication rate or poor outcome, but, in accordance with the aforementioned study outcome, the complication rate was better in patients with thoracic spinal meningiomas in comparison to those with cervical tumors. Other factors that are associated with higher complication rates are obesity, reoperation, a lack of surgeon experience and presence of calcification, and length of operation [19,20]. From the factors tested in our cohort, we found the operative time to be associated with a worsening of the functional score, which may relate to the size and complexity of the tumor. The inverse correlation between the degree of cord compression or occupation ratio and the duration of surgery found in our study supports this statement.

Experimental studies have shown that functional recovery after acute spinal cord compression depends on both the magnitude of the compression force and its duration [21,22]. Another clinical study confirmed that in acute spinal cord compression, regardless of the cause, the prognosis for recovery depends primarily on two factors: the severity of the neurological deficit and the duration of the deficit before decompression [23]. Our results showed that 84% of the patients had a better or unchanged McCormick scale result postoperatively. When comparing the measurement intervals, we found that the functional status of the patients continuously improved during the follow-up. These data demonstrate that the neurological function may improve even a long time after the decompression of the spinal cord, and stress the importance of long-term multimodal rehabilitative treatment in such cases.

Moreover, regular follow-up may be needed, not only as part of the oncological routine but also in order to monitor the patient’s progress and recovery of the neurological status.

In addition, our study showed that a worse preoperative Frankel grade can be taken as a predictor of postoperative deterioration and that patients with a worse preoperative Frankel score were up to nine times more likely to have a postoperative deterioration of functional status. 

Few studies have examined tumor volume and its impact on the severity of preoperative symptoms [8,24,25]. Our study showed significant differences in the tumor volume, tumor cross-section, occupancy ratio, and preoperative McCormick scale.

Our data are consistent with the published results from Jesse and colleagues who also demonstrated that the degree of spinal cord compression is associated with preoperative McCormick scale and the presence of sensory deficits [25].

The recurrence rate in our study was 3.3%. Similar reoperation rates have been reported in other large series [26,27,28]. Maiuri et al. reported that arachnoid invasion and higher Ki-67 could be considered significant risk factors for recurrence [29]. In our cohort, recurrence occurred in patients with calcification, multiple meningiomas, and partial resection. Given that the average time to recurrence was 5 years after surgery, long-term follow-up is needed [16].

Yamamuro et al. and Nakamura et al. found dural invasion in 76% and 35% of cases, respectively [30,31]. These results suggest that the late development of tumor recurrence is due to residual tumor cells between the inner and outer dura. In addition, the presence of a dural tail is significantly associated with a higher recurrence rate. Various other factors are associated with an increased rate of recurrence. Cohen-Gadol et al. observed recurrences in younger patients with cervical meningiomas, whereas Klekamp and Samii reported that plaque formation, infiltrating meningiomas, and arachnoid scars have a significantly higher association with increased recurrence rates [26,32]. Older patients showed a lower recurrence rate [15].

## 7. Limitations

First, this was a retrospective single-center study, which may not completely reflect the natural course of the disease. Although our patient sample was large and contained long-term follow-up data concerning functional outcomes, the number of patients with functional worsening was low, which must be taken into account when interpreting the results. 

## 8. Conclusions

According to our data, the surgery of spinal meningiomas is safe, with satisfactory results and minimal morbidity. Neurological function improved in a significant proportion of patients after surgery. Preoperative Frankel grade was a significant predictor of postoperative neurological worsening. Cross-section area measurements on MRI scans were not associated with early postoperative outcomes.

## Figures and Tables

**Table 1:** Modified McCormick scale.

Grade	Description
I	Intact neurologically, normal ambulation, minimal dysesthesia
II	Mild motor or sensory deficit, functional independence
III	Moderate deficit, limitation of function, independent with external aid
IV	Severe motor or sensory deficit, limited function, dependent
V	Paraplegia or quadriplegia, even with flickering movement

**Table 2:** Frankel scale.

Grade		Description
A	Complete	No motor or sensory function below level of lesion
B	Sensory only	No motor function, but some sensation preserved below lesion
C	Motor useless	Some motor function without practical application
D	Motor useful	Useful motor function below level of lesion
E	Recovery	Normal motor or sensory function, may have reflex abnormalities

**Table 3:** Baseline demographics and clinical characteristics.

Variable	N (%)
Gender	
Male	17 (14%)
Female	104 (86%)
Mean age ± SD, yrs	66 ± 13
Spinal level	
Cervical	36 (29.7%)
Thoracic	82 (67.7%)
Lumbar	3 (2.5%)
Clinical symptoms	
Ataxia	67 (55.4%)
Hyperreflexia	40 (33.1%)
Babinski	20 (16.5%)
Sensory deficit	83 (68.6%)
Motor deficit	58 (47.9%)
Pain	63 (52.1%)
Bladder–bowel disturbances	9 (7.4%)
Myelopathy	77 (63.6%)
Syrinx	5 (4.1%)
CNS WHO Grade	
1	113 (93.4%)
2	5 (4.1%)
Approach	
Anterior	1 (0.8%)
Posterior	117 (96.6%)
Lateral	3 (2.5%)
Complications	
Epidural hematoma	3 (2.4%)
CSF fistula	2 (1.6%)
Meningitis	1 (0.8%)
Embolism	1 (0.8%)

**Table 4:** Tumor volumetry.

Parameter	Mean	Min–Max
Volume (mm^3^)	1032.1	78.4–3126.5
Spinal canal cross-sectional area (mm^2^)	232.2	80.2–505.4
Cross-sectional area of spinal cord (mm^2^)	44.1	7.2–95.7
Cross-sectional area of tumor (mm^2^)	112.3	11.2–332.1
Occupation ratio of the tumor (%)	47.9	7.7–88.7
Degree of compression of the spinal cord (%)	29.4	4.8–83.5

**Table 5:** Correlation between tumor volumetry and McCormick scale.

	Median (IQR) McCormick					*p* *
	1	2	3	4	5	
Tumor volume (mm^3^) t2	359.4	801.1	959.8	1863.1	1500.01	<0.001 ^†^
	(227.2–834.4)	(514.5–1275.4)	(666.6–1379.3)	(878–2374)	(1226–1774)	
Cross-section (tumor) t2 (mm^2^)	47	46	81.25	109	133	<0.001 ^‡^
	(39–13)	(26–81)	(75.5–124.8)	(108–147)	(136.3–202.8)	
Cross-section (spinal canal) t2 (mm^2^)	184	217	228	297	271	0.007 ^§^
	(170–249)	(191.7–253.8)	(189.5–264.8)	(234.5–345.5)	(270–272)	
Cross-section (cord) t2 (mm^2^)	45	43	40	36	49	0.72
	(35–62)	(36–53.5)	(28.8–54.3)	(30–60.8)	(37–61)	
Occupation ratio	40.18	48	48	61.17	41.11	0.03 ^≈^
	(29.3–48.2)	(41.1–53.6)	(37.5–62.8)	(49.5–65.6)	(33.3–48.9)	
Degree of cord compression	17.6	22.64	30.15	24.1	25.9	0.13
	(14.5–24.1)	(12.3–34.8)	(16.2–46.5)	(19.3–54.7)	(15.8–36.1)	

IQR—interquartile range; * Kruskal–Wallis test (post hoc Conover). ^†^ at the *p* < 0.05 level, there are significant differences (1) vs. (2), (3), (4), (5); (4) vs. (2), (3). ^‡^ at the *p* < 0.05 level, there are significant differences (1) vs. (2), (3), (4); (4) vs. (2), (3). ^§^ at the *p* < 0.05 level, there are significant differences (4) vs. (1), (2), (3). ^≈^ at the *p* < 0.05 level, there are significant differences (1) vs. (3), (4); (2) vs. (4).

**Table 6:** Correlation between tumor volumetry and Frankel grade.

	Median (IQR) Frankel Scale					*p* *
	A	B	C	D	E	
Tumor volume (mm^3^) t2	1500	1595.8	1005.5	884.8	294.7	0.007 ^†^
	(1226–1774)	(688.1–2503.6)	(693.4–1829.2)	(523.2–1300.6)	(170–834)	
Cross-section (tumor) t2 (mm2)	111.5	172	109	109	68.5	0.03 ^‡^
	(90–133)	(143–201)	(81.5–146.5)	(77–137.3)	(49–92)	
Cross-section (spinal canal) t2 (mm^2^)	271	279,5	238	220	199.5	0.13
	(270–272)	(262–297)	(194–288.5)	(190.5–257)	(170–275)	
Cross-section (cord) t2 mm^2^	49 (37–61)	55.5 (46–65)	38 (30–53)	41 (31–54)	46 (35–62)	0.49
Occupation ratio	41.1	61.1	49.8	48	35.7	0.03 ^‡^
	(33.3–48.9)	(54.5–67.7)	(39.7–60.8)	(40.8–57.4)	(28.3–46.9)	
Degree of cord compression	25.9	36.2	28.8	22.9	15.6	0.13
	(15.9–36)	(24.6–47.8)	(17.8–49.1)	(13.9–46.3)	(14.5–23.1)	

IQR—interquartile range; * Kruskal–Wallis test (post hoc Conover). ^†^ At the *p* < 0.05 level, there are significant differences (E) vs. (A), (C), (D). ^‡^ At the *p* < 0.05 level, there are significant differences (E) vs. (B), (C), (D).

**Table 7:** Analysis according to Spearman’s correlation coefficient.

	Frankel Scale	Degree of Cord Compression	Occupation Ratio	Operative Time
Frankel scale	1			
Degree of cord compression	−0.228 (0.01)	1		
Occupation ratio	−0.173 (0.07)	0.371 (<0.001)	1	
Operative time	−0.249 (0.006)	0.211 (0.03)	0.043 (0.65)	1
Blood loss	−0.222 (0.02)	0.140 (0.14)	0.083 (0.38)	0.448 (<0.001)

**Table 8:** Multivariate logistic regression analysis for worsening of neurological function.

Parameter	Odds Ratio	95% CI	*p*-Value
Frankel Scale	9.16	3.69–22.7	<0.001
Lower thoracic spine	0.28 *	0.10–0.75	0.01
KPS	1.07	1.02–1.13	0.009
Operative time	1.02	1.003–1.04	0.02

* *p* < 0.05. The factors that were statistically significant in a univariate analysis (i.e., x, y, z….) were used for a multivariate model. Nagelkerke R square. CI—confidence interval.

## Data Availability

Data are available at the Department of Neurosurgery at the University Medical Mainz and can be requested from the director (Prof. Florian Ringel). Each request should be based on a scientific hypothesis and reviewed by a (local) ethical committee. Any request must be made in writing. Data will be saved for ten years after publishing (according to GCP-guidelines).
